# Molecular Dynamics Simulation of the Compatibility Between Supercritical Carbon Dioxide and Coating Resins Assisted by Co-Solvents

**DOI:** 10.3390/ma17246271

**Published:** 2024-12-22

**Authors:** Nan Wang, Chenxiao Pei, Yuhang Zhong, Yuqi Zhang, Xingang Liu, Jianyuan Hou, Yuan Yuan, Renxi Zhang

**Affiliations:** Shanghai Key Laboratory of Atmospheric Particle Pollution and Prevention (LAP3), Institute of Environmental Science, Fudan University, Shanghai 200433, China; wnan0415@163.com (N.W.); 22210740066@m.fudan.edu.cn (C.P.); 23210740107@m.fudan.edu.cn (Y.Z.); 23210740100@m.fudan.edu.cn (Y.Z.); xingangliu@fudan.edu.cn (X.L.); hjy@fudan.edu.cn (J.H.); yuan_y@fudan.edu.cn (Y.Y.)

**Keywords:** supercritical carbon dioxide, PVDF, co-solvent, molecular dynamics simulation, compatibility

## Abstract

The use of supercritical carbon dioxide (ScCO_2_) as a replacement for volatile organic solvents in coatings has the potential to reduce air pollution. This paper presents the findings of a molecular dynamics simulation study investigating the dissolution behavior of polyvinylidene fluoride (PVDF) in ScCO_2_ assisted by five co-solvents. On the basis of solubility parameters, interaction binding energy, and radial distribution functions, the impacts of temperature, pressure, and co-solvents on the compatibility of ScCO_2_ and PVDF were investigated at the microscopic level. The simulation results demonstrated that low-temperature and high-pressure conditions facilitate the dissolution of PVDF in ScCO_2_, where the optimal conditions are 308.15 K and 16 MPa. The enhancement of the solubility performance of ScCO_2_ slowed down with increasing pressure, but was more sensitive to changes in temperature. The weak attraction between PVDF and ScCO_2_ was synergized by van der Waals and electrostatic forces, making it challenging to achieve complete and homogeneous mixing. The use of co-solvents with strong polarity can enhance the solvent system’s solubility. Ethanol and 2-butoxy-1-ethanol have obvious solubilizing abilities due to the hydrogen bond donors, which can generate hydrogen bonding interactions with ScCO_2_, increase the polarity of the solvent system, and promote the compatibility of ScCO_2_ with PVDF.

## 1. Introduction

The coatings industry is an important source of volatile organic compounds (VOCs). Replacing or reducing the use of organic solvents in coatings is a hotspot of concern for VOC reduction in this field [1,2]. Supercritical carbon dioxide (ScCO_2_) is used as an excellent solvent with characteristics similar to the high diffusivity of a gas and the solubility of a liquid [3,4,5]. The use of ScCO_2_ to replace volatile organic solvents in coatings is a highly promising and environmentally friendly approach [6,7].

Despite the good solvent properties of ScCO_2_, its weak affinity for polymers leads to a limitation of the solubilization of resins in coatings by ScCO_2_, due to the fact that CO_2_ is a non-polar molecule with a zero permanent dipole moment [8,9,10]. Therefore, it is particularly important to carry out studies to enhance the compatibility of resin with ScCO_2_ and the related dissolution mechanism.

Adding co-solvents is an effective way to improve the compatibility of resins with ScCO_2_ [11,12,13]. However, the selection of co-solvents and the exploration of the mechanism are often constrained by several factors. Experimental methods are often limited by human factors, operational precision, and the difficulty of controlling conditions. This makes it difficult to establish a theoretical foundation and to make effective predictions regarding the selection of co-solvents. Molecular dynamics (MD) simulation is a significant method for studying supercritical fluids, offering a means to overcome the limitations of experimental studies and facilitating comprehensive theoretical analyses [14,15,16]. Nevertheless, MD simulations related to ScCO_2_ have mainly focused on its interaction with small molecules, and little research has been reported on the interaction between coating resins and ScCO_2_ assisted by co-solvents.

In this study, the dissolution behavior of polyvinylidene fluoride (PVDF) and ScCO_2_ was investigated using MD simulations with the assistance of five co-solvents: acetone (AC), o-xylene (O-X), *n*-butyl acetate (BA), ethanol (ET), and 2-butoxy-1-ethanol (BE). The impacts of temperature, pressure, and co-solvents on the compatibility of ScCO_2_ and PVDF were investigated through the calculation of the solubility parameter [17], interaction binding energy [18], and radial distribution function (RDF) [19]. Furthermore, the effects of different co-solvents on improving the compatibility of ScCO_2_ and PVDF were compared. The mechanisms by which co-solvents enhance the compatibility of ScCO_2_ and PVDF are explained.

## 2. Methods of Analysis

### 2.1. Simulation Details

**Figure 1:**
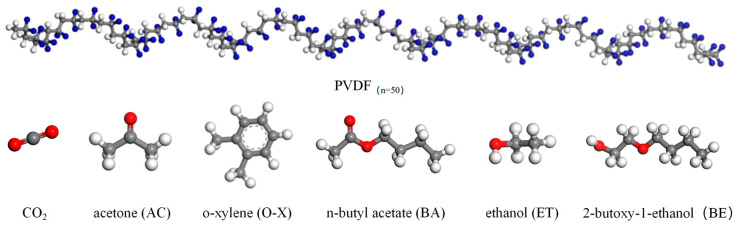
Molecular structures of CO_2_, PVDF, and different co-solvents.

**Figure 2:**
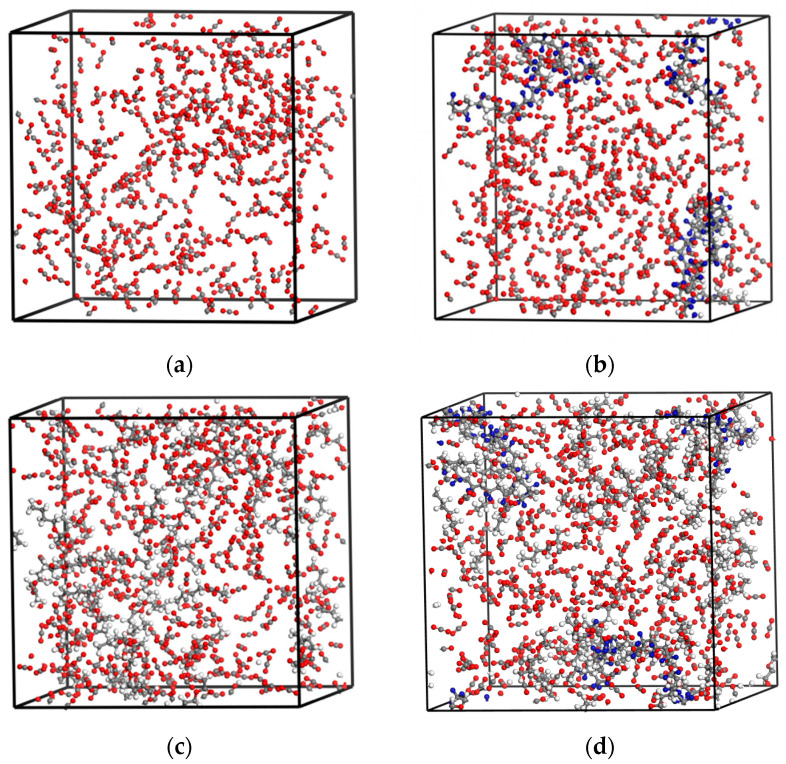
Amorphous cells of different solvation systems: (**a**) CO_2_; (**b**) PVDF/CO_2_; (**c**) CO_2_/co-solvent; (**d**) PVDF/CO_2_/co-solvent.

In this work, all calculations were performed using Material Studio 2019 software (Accelrys USA Inc., San Diego, CA, USA). First, molecular models of CO_2_, PVDF, and co-solvents were established. Once the degree of polymerization reaches a certain value, the physical parameters of the polymer stabilize [20,21]. Therefore, the repeating units of PVDF were set to 50, which corresponds to a molecular weight of 3200 g/mol. Their structures were optimized using the smart minimizer algorithm with a calculation accuracy of 1 × 10^−3^ kcal/mol. The structures of the optimized models are shown in Figure 1. In order to obtain thermodynamic parameters that are as realistic as possible while avoiding excessive calculation time, the following settings were applied to the system composition, as detailed in Table 1. Periodic boxes were constructed using the Amorphous cell module for four types of systems: CO_2_, PVDF/CO_2_, CO_2_/co-solvent, and PVDF/CO_2_/co-solvent, as shown in Figure 2.

Molecular dynamics simulations of the aforementioned systems were conducted using the COMPASS force field, which is based on the optimization of experimental data and ab initio calculations and has been widely recognized [22]. The COMPASS force field is suitable for small-molecule CO_2_ and polymer systems [19,23].

The configuration with the lowest energy was selected as the initial system. This system was then annealed for five cycles with the objective of eliminating local irrational structures. The annealing process was conducted from 300 to 500 K and then back to 300 K, with a total of 5000 steps. Subsequently, the annealed configuration was set under an NVT ensemble with a time step of 1 fs for 100 ps to ensure that the system was maintained at the correct temperature. The temperature was controlled using a Berendsen thermostat [24]. Finally, the MD simulation was employed in an NPT ensemble with 400 ps. The temperature was regulated using an Andersen thermostat, and the pressure was controlled using a Berendsen thermostat. The time step was 1 fs, and the trajectories were output every 5 ps. During the simulation process, the system’s potential energy and density reached a steady state at 350 ps, while the temperature and pressure remained at the set values, indicating that the system was in equilibrium. The final 50 ps configurations were then utilized for statistical analysis. The Lennard-Jones function and the Coulombic equation were used to calculate the non-bond interactions, which consist of long-range electrostatic interactions and short-range van der Waals interactions [25]. The Ewald summation method was used for electrostatic interactions and atom-based van der Waals interactions with a cut-off radius of 15.5 Å [26].

**Figure 3:**
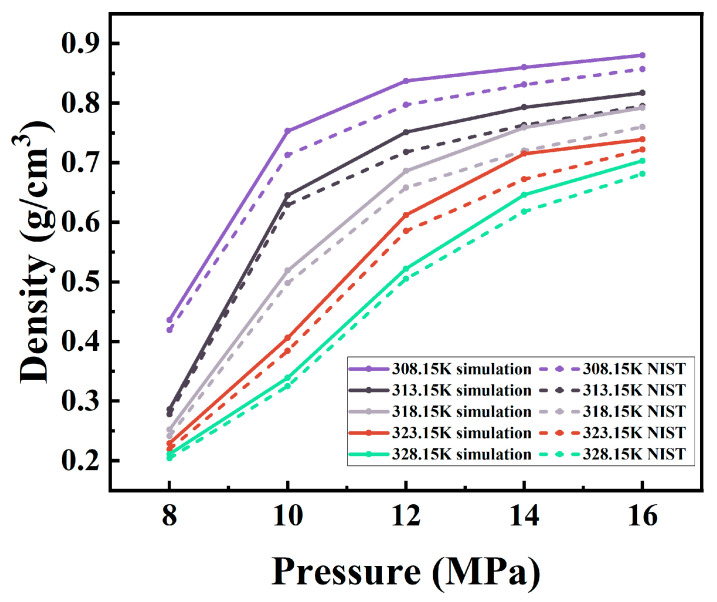
Comparison of the simulated and NIST values of ScCO_2_ density.

In order to validate the applicability of the COMPASS force field and the reliability of the simulation method, MD simulations of the density of ScCO_2_ were performed at temperatures of 308.15, 313.15, 318.15, 323.15, and 328.15 K, and pressures ranging from 8 to 16 MPa. As the simulation time increased, the system reached equilibrium, and the density of ScCO_2_ became stable. The data obtained were compared with the National Institute of Standards and Technology (NIST) database [27], as shown in Figure 3. The simulation results are largely in agreement with the NIST data, with a relative error range of 2.35% to 6.39%. This provides evidence of the validity of the COMPASS force field and the simulation method.

### 2.2. Parameter Selection

(1) Solubility parameter

The solubility parameter is a pivotal characteristic parameter for investigating the compatibility between a solvent and solutes. The closer the solubility parameter of the solutes is to that of ScCO_2_, the greater the compatibility between the two substances [28]. The solubility parameter is defined as the half-power of cohesive energy density (CED). The latter is understood to be the energy associated with molecular interactions. The equation is as follows [29]:

(1) $$\mathrm{CED}\;\;\frac{\Delta H_{v}-RT}{V_{m}}=\frac{E_{coh}}{V_{m}}\;\delta =\sqrt{CED}=\sqrt{\frac{\Delta H_{v}-RT}{V_{m}}}$$

where Δ*Hv* is the molar heat of evaporation, kJ/mol; *RT* is the expansion work carried out during gasification, kJ/mol; *Vm* is the molar volume, mol/L; *Ecoh* is defined as the average energy required to separate all molecules over an infinite distance; *CED* is the cohesive energy density, J/m^3^; and *δ* is the solubility parameter, (J/m^3^)^1/2^.

(2) Binding energy

The interaction binding energy is used to describe the energy required to separate two substances, and it represents the strength of the interaction between the two substances. In general, the larger the binding energy, the stronger the intermolecular interaction force and the better the compatibility between the two substances [30]. To gain a deeper understanding of the interaction strength between PVDF and solvents (ScCO_2_ or ScCO_2_ + co-solvents), the binding energy was calculated. The binding energy was determined using the following equation [31]:

*E*_bind_ *= −E*_inter_ *= (E*_solvents_ *+ E*_PVDF_*) − E*_solvents_+_PVDF_ (2)

where *E*_bind_ and *E*_inter_ are the binding energy and interaction energy between PVDF and solvents, kJ/mol; *E*_solvents_ is the energy of solvents, kJ/mol; *E*_PVDF_ is the energy of PVDF, kJ/mol; and *E*_solvents_+_PVDF_ is the total energy of the system, kJ/mol.

(3) Radial distribution function

The radial distribution function (RDF) is a physical quantity that reflects the characteristics of molecular aggregation and the regularity of interactions within a system. It shows the proportion of the probability of a second particle appearing at distance r around a particle central to its proportion in the case of a random distribution [32]. The type of interaction can be determined from the peak position of the RDF, and the magnitude of the interaction force can be estimated through the height of the peak. Generally, short-range interactions include hydrogen bonding (<3.5 Å) and van der Waals interactions (3.5–5.0 Å). Long-range interactions are usually referred to as electrostatic interactions (>5.0 Å) [5]. The RDF calculation equation is as follows [19]:

(3) $$\begin{array}{ll} g_{AB}\left(r\right) & =\frac{1}{\rho_{AB}\cdot \frac{4\pi }{3}\left[{\left(r+\delta r\right)}^{3}-r^{3}\right]}\frac{\sum_{i=1}^{T}\sum_{j=1}^{N_{AB}}\Delta N_{AB}\left(r\to r+\delta r\right)}{N_{AB}\cdot T}\\ & \approx \frac{1}{\rho_{AB}4\pi r^{2}\delta r}\frac{\sum_{i=1}^{T}\sum_{j=1}^{N_{AB}}\Delta N_{AB}\left(r\to r+\delta r\right)}{N_{AB}\cdot T}\left(\delta r\to 0\right) \end{array}$$

where $g_{AB}\left(r\right)\;$ denotes the probability of B particle occurring in the range r to *r* + δr centered on *A* particle; Δ*N*_AB_ is the number of *A* (or *B*) from *r* to *r* + *δr* around *B* (or *A*); $\rho_{AB}$ is the density of the whole system; *N_AB_* stands for the total number of *A* and *B*; and *T* stands for the total simulation time.

## 3. Results and Discussion

### 3.1. Comparison of the Solubility Parameters of PVDF and ScCO_2_ with Co-Solvent Assistance

**Figure 4:**
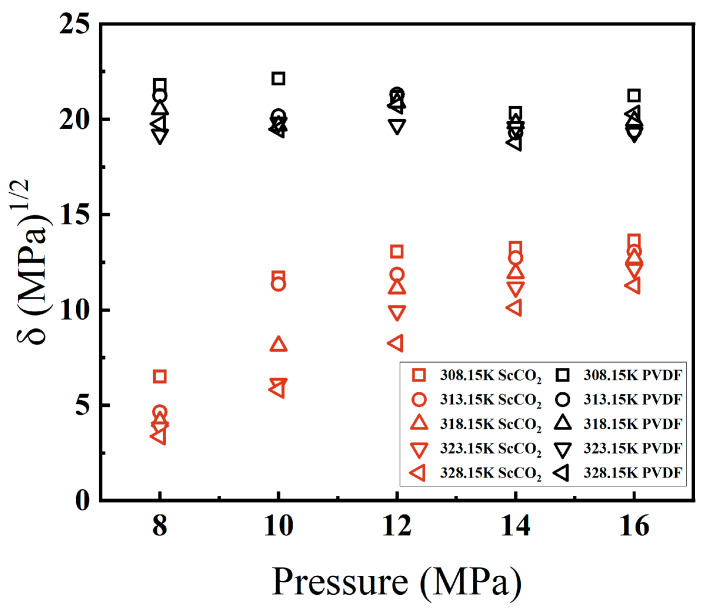
Variation of solubility parameters of PVDF and ScCO_2_ with temperature and pressure.

The solubility parameters of ScCO_2_ and PVDF at temperatures of 308.15, 313.15, 318.15, 323.15, and 328.15 K and pressures in the range of 8–16 MPa were calculated through MD simulation, as illustrated in Figure 4. As shown in Figure 4, the solubility parameter of PVDF demonstrates greater stability, with temperature and pressure fluctuations exerting a relatively minimal influence on its solubility parameter. In the case of ScCO_2_, it was observed that as the temperature remained constant, an increase in pressure resulted in a corresponding rise in the solubility parameter. Furthermore, it was noted that this increase in solubility parameter exhibited a tendency toward stability with a rise in pressure. When the pressure was held constant, the solubility parameter of ScCO_2_ exhibited a decrease with increasing temperature. The solubility parameter of ScCO_2_ reached its maximum at 308.15 K and 16 MPa, and the solubility parameters of ScCO_2_ and PVDF were the most closely aligned. Nevertheless, the discrepancy between the two remained considerable, indicating that complete and uniform mixing of the two is challenging, which aligns with the limited solubility of ScCO_2_ for macromolecules.

**Figure 5:**
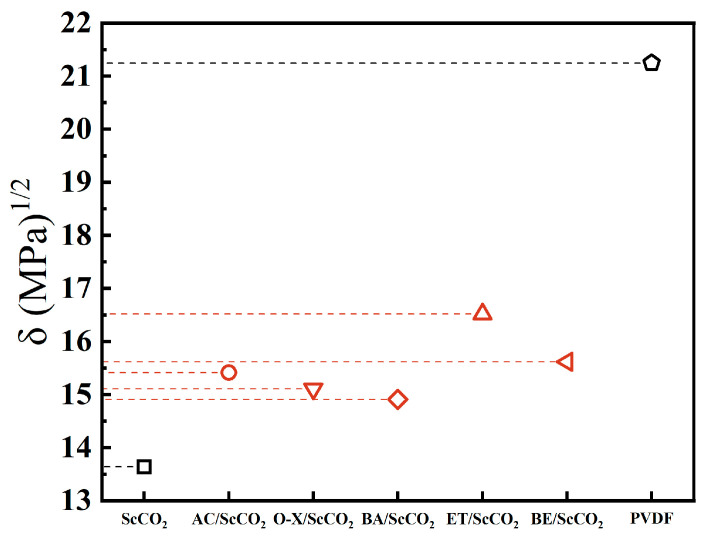
Comparison of the solubility parameters between PVDF and ScCO_2_/co-solvent.

At a temperature of 308.15 K and a pressure of 16 MPa, co-solvents with a mass fraction of 20% were introduced into the ScCO_2_ system. The co-solvents included acetone (AC), o-xylene (O-X), *n*-butyl acetate (BA), ethanol (ET), and 2-butoxy-1-ethanol (BE). The solubility parameters of these co-solvents were then compared to those of PVDF, as illustrated in Figure 5. It is evident that the utilization of different co-solvents can enhance the solubility parameter of the solvent system, thereby facilitating the solubility of ScCO_2_; however, the degree of solubilization exhibited considerable variation. In descending order, the solubility parameters of the solvent system were improved by ET, BE, AC, O-X, and BA. From the perspective of polarity, ET, BE, and AC are classified as polar solvents. They are capable of effectively enhancing the polarity of the system and increasing the intermolecular interaction force, thereby improving the system’s solubility parameters [4].

### 3.2. Comparison of the Interaction Binding Energies of PVDF and ScCO_2_ with Co-Solvent Assistance

**Figure 6:**
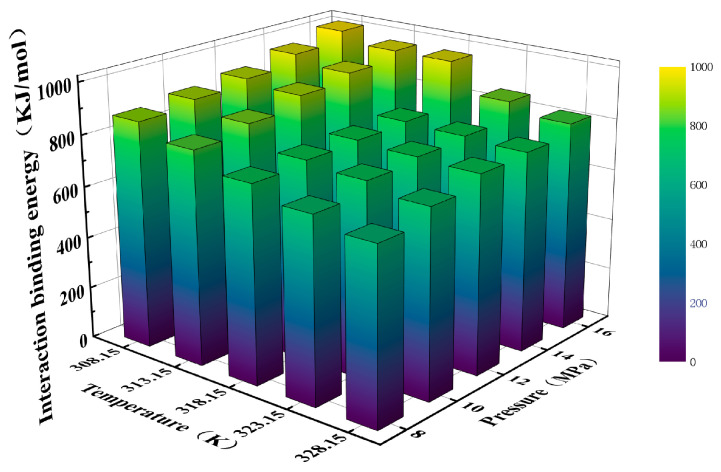
The binding energy of ScCO_2_ and PVDF at different temperatures and pressures.

The binding energy values of ScCO_2_ and PVDF at temperatures of 308.15, 313.15, 318.15, 323.15, and 328.15 K and pressures ranging from 8 to 16 MPa were calculated using molecular dynamics (MD) simulation, as illustrated in Figure 6. At a temperature of 328.15 K and a pressure of 8 MPa, the binding energy of PVDF and ScCO_2_ was 669.57 kJ/mol. In contrast, at a temperature of 308.15 K and a pressure of 16 MPa, the binding energy of the two reached 1006.62 kJ/mol. As the temperature decreased and the pressure increased, the binding energy of PVDF and ScCO_2_ increased by 50.33%, which aligns with the solubility parameters. Table 2 illustrates the contribution of each energy term to the binding energy when the system was set to 308.15 K and 16 MPa. The data in the table demonstrate that the interaction energy between PVDF and ScCO_2_ arises from non-bonding energy, indicating that no chemical bond is formed, which is consistent with the observed phenomenon. The non-bonding energy was primarily constituted by van der Waals and electrostatic forces, with the van der Waals force energy term contributing 597.81 kJ/mol, slightly exceeding the contribution of the electrostatic force energy term (381.51 kJ/mol). Furthermore, it can be observed that the binding energy of ScCO_2_ with PVDF (1006.62 kJ/mol) was considerably smaller than that of the PVDF molecule itself (12137.11 kJ/mol), indicating that the two are only weakly attracted to each other. This implies that ScCO_2_ is insufficient to prevent the interaction of the PVDF molecule itself, which provides a potential explanation for the limited dissolution behavior of macromolecules in ScCO_2_ observed in previous studies.

**Figure 7:**
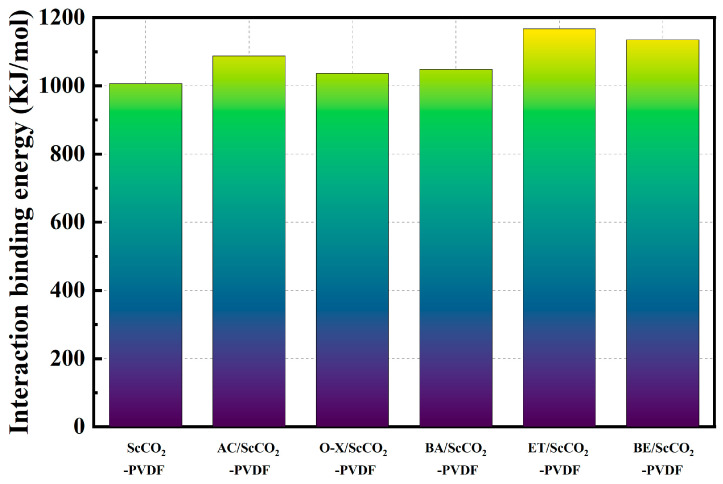
Binding energy between ScCO_2_/co-solvent and PVDF.

The impact of the addition of the aforementioned five co-solvents on the binding energy was evaluated at 308.15 K and 16 MPa. As illustrated in Figure 7, the impact of varying co-solvent concentrations on the interaction energy between PVDF and the solvent system exhibited notable disparities. The order of decreasing intensity of the interaction energy between the solvent system and PVDF is as follows: ET/ScCO_2_, BE/ScCO_2_, AC/ScCO_2_, BA/ScCO_2_, and O-X/ScCO_2_. The magnitude of this intensity is consistent with the magnitude of the solubility parameter, indicating that intermolecular interactions are a significant factor influencing the solubility parameter. Of the aforementioned five co-solvents, AC, ET, and BE were more polar. Among these, ET and BE demonstrated a superior solubilization effect. In a system lacking co-solvents, the binding energy between PVDF and ScCO_2_ was calculated to be 1006.62 kJ/mol. The addition of ET resulted in an increase in binding energy to 1067.19 kJ/mol, which may be attributed to its capacity to form hydrogen bonds with ScCO_2_ and thereby enhance intermolecular interactions. BA exhibited a lower level of polarity than the aforementioned three solvents, yet its improvement in intermolecular solubilization effect was slightly more pronounced than that of O-X.

### 3.3. Comparison of Radial Distribution Functions of PVDF and ScCO_2_ with Co-Solvent Assistance

**Figure 8:**
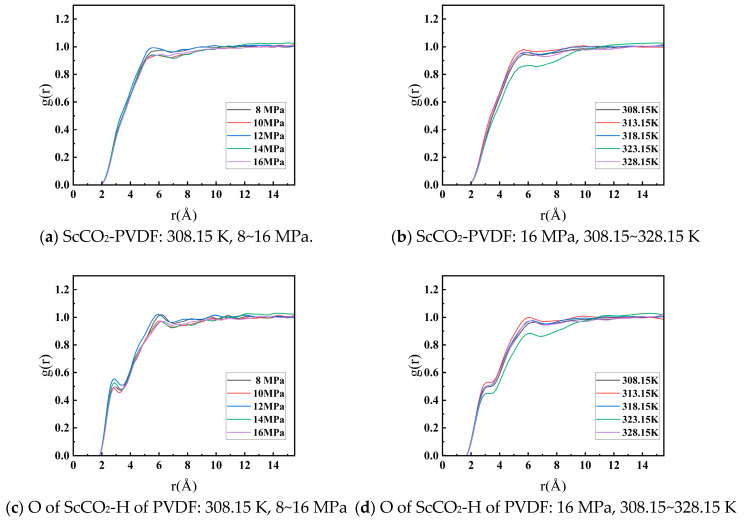
Comparison of the radial distribution function of ScCO_2_-PVDF and O of ScCO_2_-H of PVDF.

The radial distribution functions (g(r)) of ScCO_2_ and PVDF molecules, O atoms of ScCO_2_, and H atoms of PVDF were analyzed at 308.15 K and 8–16 MPa, as well as at 16 MPa and a temperature range of 308.15–328.15 K. From Figure 8a,b, it can be observed that the g(r) distribution curves of ScCO_2_ and PVDF at varying temperatures and pressures exhibited a comparable pattern, with peaks occurring at approximately 5 Å. This indicates the presence of intermolecular aggregation, with the peak positions suggesting that the intermolecular interactions are governed by a combination of van der Waals and electrostatic forces. Figure 8a illustrates that the position of the peak remained relatively constant when the pressure was altered. The data indicate that pressure has no significant effect on the interaction distance between ScCO_2_ and PVDF molecules. From Figure 8b, it can be observed that the position of the peak exhibited a slight rightward shift with increasing temperature. This indicates that elevated temperatures result in a longer interaction distance between ScCO_2_ and PVDF molecules, leading to a reduction in the binding energy of intermolecular interactions. This suggests that temperature fluctuations exert a more pronounced influence on the dissolution characteristics of ScCO_2_ than on pressure fluctuations. Furthermore, Figure 8c,d illustrate that the g(r) of the O atoms of ScCO_2_ and the H atoms of PVDF exhibited a peak between 2 and 3 Å. Given that the hydrogen atoms on the PVDF molecule chain lack strong electronegative atoms (such as N, O, and F) in their vicinity, it was initially postulated that weak synergistic hydrogen bonding formed between the two, thereby reinforcing the interaction between ScCO_2_ and PVDF to a certain extent.

**Figure 9:**
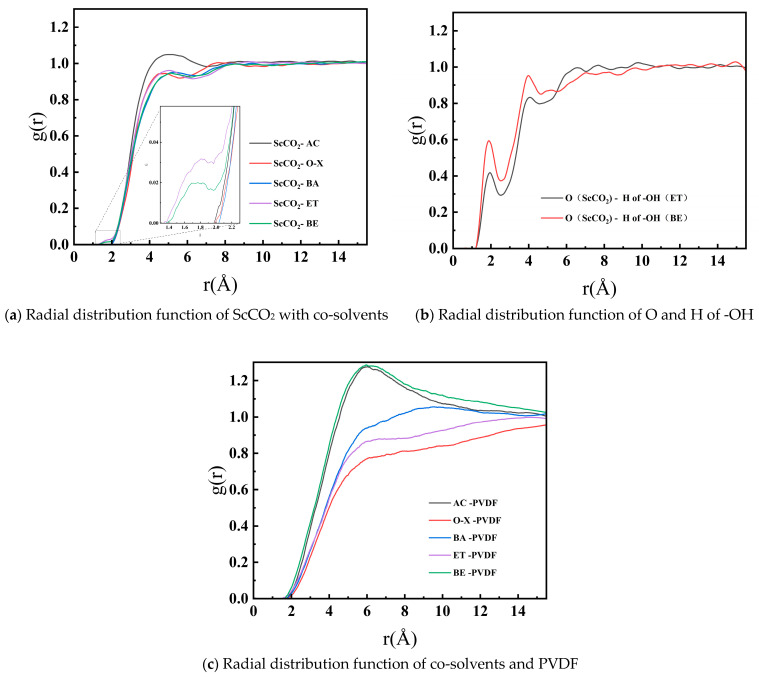
Radial distribution function between ScCO_2_, co-solvents, and PVDF and special atom pairs.

The radial distribution functions (g(r)) of ScCO_2_/co-solvent, as well as co-solvent/PVDF, were calculated at 308.15 K and 16 MPa, respectively. Figure 9a illustrates the presence of pronounced peaks at 4–6 Å between ScCO_2_ and all five co-solvents, indicative of an aggregation phenomenon between ScCO_2_ and the aforementioned co-solvent molecules. The maximum peak value did not exceed 1.2, substantiating the assertion that the interaction between ScCO_2_ and the co-solvents was relatively weak. This can be attributed to the combined influence of van der Waals and electrostatic forces. The peak height indicates that the aggregation phenomenon produced by AC molecules with ScCO_2_ was the most pronounced, which is attributed to the strong polarity of AC. Figure 9a also demonstrates that the radial distribution function g(r) of ScCO_2_-ET, as well as ScCO_2_-BE, exhibited pronounced peaks at 1.6–2.0 Å, which is somewhat distinct from that of other solvents. This suggests the potential for a two-layer arrangement of intermolecular distribution. To ascertain whether ET and BE engage in hydrogen bonding interactions with ScCO_2_, the radial distribution functions g(r) of the H atoms on the hydroxyl groups of both and the O atoms of ScCO_2_ were analyzed, respectively, as illustrated in Figure 9b. Figure 9b shows that g(r) exhibited a pronounced peak at approximately 2.0 Å, indicating that the ScCO_2_-ET and ScCO_2_-BE solvent systems formed more robust hydrogen bonds. This led to a notable enhancement in the solubility parameter of the solvent system, which in turn improved its compatibility with PVDF. As illustrated in Figure 9c, the interactions between different co-solvents and PVDF were predominantly governed by electrostatic forces with long-range interactions. This is evidenced by the pronounced aggregation observed between AC, BE, and PVDF, which suggests that polar co-solvents interact more strongly with PVDF.

### 3.4. Mechanism Analysis of the Effect of Co-Solvent-Assisted Compatibility of PVDF with ScCO_2_

Based on the aforementioned results, the solubility parameters, interaction binding energies, and radial distribution functions of PVDF and ScCO_2_ exhibited consistent patterns in response to changes in temperature and pressure. This is due to the fact that alterations in temperature and pressure result in a modification of the intermolecular interaction distance, which influences the magnitude of the interaction strength. This is manifested as a change in the solubility parameter overall. It was observed that as the temperature remained constant, the solubility of ScCO_2_ in PVDF increased in conjunction with the rise in pressure. An increase in system pressure resulted in an increase in the density of ScCO_2_ and a reduction in the intermolecular distance of the system. This led to an enhancement of the interaction force with PVDF and an increase in the binding energy between ScCO_2_ and PVDF. As the pressure increased, the compressibility of ScCO_2_ diminished, and the rise in interaction strength with PVDF became less pronounced. It was observed that when the pressure was held constant, the solubility of ScCO_2_ in PVDF decreased with an increase in temperature. This is due to the intensification of molecular thermal movement in the system resulting from the increase in temperature. This led to an increase in the interaction distance between ScCO_2_ molecules and PVDF, a weakening of the intermolecular interaction force, and a subsequent decrease in the binding energy of ScCO_2_ and PVDF. It can be observed that maintaining the system temperature at a slightly elevated level above the critical conditions for ScCO_2_ and increasing the system pressure facilitated the dissolution of PVDF in ScCO_2_; however, the enhancement of the dissolution performance of ScCO_2_ by elevated pressure was limited. In actual fact, several studies have verified that fluorinated polymers exhibit better solubility in ScCO_2_ than other polymers [33,34,35]. The O atom of CO_2_ exhibits a slight negative charge, which enables it to form weak cooperative hydrogen bonding with the H atom of PVDF and also to engage in Lewis acid–base interactions with CO_2_ [36]. This enhances the affinity between the two. This provides an explanation of the results presented in Figure 8c,d, showing a peak between 2 and 3 Å in g(r).

The addition of co-solvents was demonstrated to enhance the compatibility between ScCO_2_ and PVDF. The results demonstrated that the solubilizing effect of the co-solvents descended in the order of ET, BE, AC, O-X, and BA. This is attributed to the differing dipole moments of the co-solvents, which exhibited varying polar strengths and weaknesses. Consequently, the polarity of the solvent system was enhanced to varying degrees, thereby optimizing the solubilizing performance of the solvent system. It can be observed that the solubilizing effect was directly proportional to the polarity of the co-solvents. Among the co-solvents, AC exhibited the largest dipole moment (2.9 D) and the strongest polarity. However, its solubilizing effect was less pronounced than that of ET and BE. This can be attributed to the synergistic effect of van der Waals and electrostatic forces between ET and BE, as well as the generation of hydrogen bonds, which effectively enhanced the polarity of the solvent system. Consequently, an increase in the polarity of the co-solvents resulted in an increase in the occurrence of aggregation phenomena, with AC and BE exhibiting the most pronounced effects. This is in accordance with the general principle that polar solutes are readily soluble in polar solvents. Accordingly, when selecting co-solvents, it is advisable to opt for those with elevated polarity and the capacity to form hydrogen bonds, with the objective of enhancing the compatibility between ScCO_2_ and PVDF.

## 4. Conclusions

The effects of different co-solvents on the compatibility between PVDF and ScCO_2_ were investigated using MD simulations with a focus on solubility parameters, interaction binding energy, and radial distribution functions. The main conclusions are as follows:

The weak attraction between PVDF and ScCO_2_ was synergized by van der Waals and electrostatic forces, making it challenging to achieve complete and homogeneous mixing. The addition of co-solvents led to enhancements in the compatibility between PVDF and ScCO_2_; the order of the observed enhancement effects was as follows: ethanol > 2-butoxy-1-ethanol > acetone > o-xylene > *n*-butyl acetate. Due to the formation of hydrogen bonds with ScCO_2_, the solubilizing abilities of ethanol and 2-butoxy-1-ethanol were more pronounced than that of acetone, despite acetone’s greater polarity.

The polarity of the co-solvent and its capacity to generate hydrogen bonding are pivotal in influencing the solubilizing ability. The selection of a co-solvent with greater polarity and hydrogen bonding donors can facilitate the dissolution of PVDF in ScCO_2_.

## Figures and Tables

**Table 1:** The composition of the systems.

System	Composition	Number of CO_2_	Number of Chains	Number of Co-Solvents
1	CO_2_	400	-	-
2	PVDF/CO_2_	400	1	-
3	CO_2_ + 20 wt% AC	400	-	76
4	CO_2_ + 20 wt% O-X	400	-	42
5	CO_2_ + 20 wt% BA	400	-	38
6	CO_2_ + 20 wt% ET	400	-	96
7	CO_2_ + 20 wt% BE	400	-	38
8	PVDF/CO_2_ + 20 wt% AC	400	1	76
9	PVDF/CO_2_ + 20 wt% O-X	400	1	42
10	PVDF/CO_2_ + 20 wt% BA	400	1	38
11	PVDF/CO_2_ + 20 wt% ET	400	1	96
12	PVDF/CO_2_ + 20 wt% BE	400	1	38

**Table 2:** This binding energy of PVDF with ScCO_2_ at 308.15 K and 16 MPa (kJ/mol).

	System	PVDF/ScCO_2_	ScCO_2_	PVDF	E_inter_
Energy					
Total		−14,769.23	−1625.49	−12,137.11	−1006.62
Non-bond energy		−13,846.47	−3660.42	−9179.42	−1006.62
van der Waals		−2360.47	−2084.57	321.91	−597.81
Electrostatic		−11,377.42	−1496.93	−9498.97	−381.51

## Data Availability

The original contributions presented in this study are included in the article. Further inquiries can be directed to the corresponding author.
